# Linking Public Health, Housing, and Indoor Environmental Policy: Successes and Challenges at Local and Federal Agencies in the United States

**DOI:** 10.1289/ehp.8990

**Published:** 2007-01-25

**Authors:** David E. Jacobs, Tom Kelly, John Sobolewski

**Affiliations:** 1 National Center for Healthy Housing, Columbia, Maryland, USA; 2 U.S. Environmental Protection Agency, Washington, DC, USA; 3 Cuyahoga County Board of Health, Cleveland, Ohio, USA

**Keywords:** built environment, healthy housing, housing, indoor air quality, indoor environmental quality, policy, public health

## Abstract

We describe the successes and challenges faced by federal and local government agencies in the United States as they have attempted in recent years to connect public and environmental health, housing, community development, and building design with environmental, housing, and building laws, codes, and policies. These policies can either contribute to or adversely affect human physical and mental health, with important implications for economic viability, research, policy development, and overall social stability and progress. Policy impediments include tension between housing affordability and health investment that causes inefficient cost-shifting, privacy issues, unclear statutory authority, and resulting gaps in responsibility for housing, indoor air, and the built environment. We contrast this with other environmental frameworks such as ambient air and water quality statutes where the concept of “shared commons” and the “polluter pays” is more robust. The U.S. experiences in childhood lead poisoning prevention, indoor air, and mold provide useful policy insights. Local programs can effectively build healthy homes capacity through local laws and housing codes. The experience of coordinating remediation for mold, asthma triggers, weatherization, and other healthy housing improvements in Cuyahoga County, Ohio, is highlighted. The U.S. experience shows that policymakers should adopt a prevention-oriented, comprehensive multi-disciplinary approach at all levels of government to prevent unhealthy buildings, houses, and communities.

## Introduction

This article examines policy challenges faced by federal and local governments in the United States as they address the adverse health consequences of substandard housing, buildings, and other indoor environments. Specific experiences at the U.S. Department of Housing and Urban Development (HUD), the U.S. Environmental Protection Agency (EPA), and the Cuyahoga County Health Department (near Cleveland, Ohio) are highlighted as examples of how multidisciplinary approaches can yield important advances in environmental health in an area in which both scientific research and legal authorities remain relatively underdeveloped compared with existing building code, environmental law, and public health practice.

There is renewed interest in the linkage between substandard housing, poor indoor environmental quality, and unsustainable patterns of community development on the one hand and the state of the public’s health on the other (HUD 1999a; Sharfstein and Sandel 1998). Sustainability, affordability, and health are all highly related and dependent on one another. At the most basic level, substandard housing and buildings are unhealthy, unsustainable, and unaffordable (Jacobs 2005; Sandel et al. 2004).

### Origins of the health and housing connection

Linking housing and health is not fundamentally a new idea. Florence Nightingale said “The connection between health and the dwelling of the population is one of the most important that exists” (Lowry 1991). There is little doubt that improvements in housing in developed countries have greatly advanced the public health. Early housing standards provided for improved ventilation, sanitation, reduced crowding, structural soundness, lighting, and other habitability criteria, partly as a response to the appearance of concentrated slum housing around factories and big cities during the industrial revolution [Riis 1890; Centers for Disease Control and Prevention (CDC) 1976]. The public health movement and the housing movement have common roots a century ago in the sanitation movement that worked to clean up squalid conditions in housing. For example, the provision of indoor plumbing, still lacking in much of the developing world, had much to do with improved sanitation and the control of cholera and other similar diseases in the developed world. Yet today housing, health, and environment are all seen as separate and unrelated disciplines (HUD 1999a; Krieger and Higgins 2002; Lowry 1991).

In the developed world, housing deficiencies still exist, although the context and certain disease outcomes have changed (Matte and Jacobs 2000). Inadequate ventilation and crowding in housing contributed to the tuberculosis epidemic a century ago in the United States (Stein 1950) and remains a significant problem in developing countries today, causing 2 million deaths in 2002 [World Health Organization (WHO) 2004]. Although the tuberculosis problem seemed insurmountable, improvements in lighting, fresh air, and crowding in housing all helped to reduce its prevalence. Today, inadequate ventilation and moisture management in housing still contributes to asthma, mold-induced illnesses, carbon monoxide poisoning, and other diseases and injuries (Krieger and Higgins 2002; Matte and Jacobs 2000).

Although the focus of this mini-monograph is on housing conditions in Europe and the United States, it is worth noting at the outset that substandard housing conditions are truly a global problem. A recent United Nations report shows that more than one-third of all urban dwellers live in slums in developing countries (nearly 1 billion people), and the percentage is increasing rapidly, with the number of slum dwellers expected to double by 2030 (United Nations 2003).

Some of the key policy impediments in this field involve the tension between housing affordability and health, privacy issues, lack of clarity in statutory authority, and gaps in responsibility for the built and indoor environments.

### Indoor air pollution and the “shared commons” problem

In the United States, the Clean Air Act (1970) authorizes an intricate system of regulation and oversight to support outdoor air quality and a network of top-down mandates that originates with national legislation and standard setting and extends to states, counties, and localities for execution and monitoring. Generally, the system enjoys broad national support for clean and healthy outdoor air. Yet no such consensus has formed to support the quality of air indoors, where people do more of their breathing.

Indoor air pollution is one of the top four environmental health risks identified by the U.S. EPA and the Scientific Advisory Board authorized by Congress to consult with the U.S. EPA on technical matters (U.S. EPA 1990). On average, U.S. citizens spend about 90% of their time indoors, where indoor levels of pollutants may be two to five times higher than outside, and occasionally 100 times higher. Indoor pollution is estimated to cause thousands of cancer deaths and hundreds of thousands of respiratory health problems each year. Millions of children have experienced elevated blood levels of contaminants resulting from their exposure to indoor pollutants (U.S. EPA 1997). Other health effects include irritation of the eyes, nose, and throat, and more subtle neurotoxicologic and behavioral and other adverse effects.

In addition to public health concerns, indoor air pollution accounts for substantial economic costs. Cost–benefit estimates by the U.S. EPA (2001b) suggest that net avoidable costs associated with indoor air pollution amount to well over $100 billion annually, and more likely between $150 billion and 200 billion (all dollar amounts in this article are calculated in U.S. dollars). About 45% of those costs are attributable to avoidable deaths from radon and environmental tobacco smoke, about 45% from lost productivity, and about 10% from avoidable respiratory diseases.

Indoor air pollution can be defined as chemical, physical, or biological contaminants in the breathable air inside a habitable structure or conveyance, including workplaces, schools, offices, homes, and vehicles (the indoor environment). Indoor air pollution includes the following:

- combustion by-products;
- ozone;
- allergens (including mold spores);
- volatile organic compounds and particulate matter;
- paints, finishes, furnishings, adhesives, caulks, and pressed wood products found in building materials;
- cleaning products, personal care products, air fresheners, pesticides commonly used indoors;
- tobacco smoking, hobbies, cooking, and other occupant activities, including bringing home dry-cleaned clothes;
- bioeffluents; and
- soil gas intrusion (e.g., radon).

To address indoor air quality problems in the United States, national, state, and local legislatures have typically authorized public agencies to conduct voluntary programs intended to raise the public’s awareness about indoor air problems and appropriate actions they can take, alongside mandatory regulatory programs for ambient exterior pollution. The impetus for such contrasting public policies may be found in historical, attitudinal, and technical considerations that may have helped to inhibit the formation of a broad social and political consensus for controls on indoor air quality problems.

Historically, the legal structure for the environmental movement in the United States stands on two fundamental principles of English common law: “Shared Commons” and “The Polluter Pays” (Hardin 1968). Shared Commons is derived from medieval practice governing community use of a public resource. Although everyone’s cattle may graze on the common green, nobody’s cattle may overgraze the resource and deprive others of its use. In its current legal application, this principle means the community may act to protect its interest if private activity deprives the public of its right and reliance on a shared resource, for example, breathable exterior air. The Polluter Pays principle holds that it is the originator of the pollution, not the injured public, who bears responsibility for the cost of its control.

Against this intellectual and legal backdrop, in the late 1960s, a number of historical developments and emotionally charged episodes fed growing public outrage against the increasingly obvious outdoor pollution. For instance, in 1969, the Cuyahoga River in Cleveland, Ohio, burst into flames because of the combustible waste it was carrying routinely. Although the fires were quickly doused, the spectacle was dramatic enough to generate national press coverage of exterior environmental degradation in many other sectors of American national life. With respect to exterior air quality, around the same time, private charities called “Fresh Air Funds” were widely supported because of their promise to send urban children to camp for a week, allowing some to escape, if only for a brief interlude, the increasing choke of urban exterior air pollution.

With such incidents firing the revulsion of common citizens to increasingly visible environmental insults, the first Earth Day in 1970 brought millions into the streets to express broad and deep public outrage over the problem. President Richard Nixon responded by establishing the U.S. EPA by Executive Order in December 1970 (Office of the President 1970). By that time, the U.S. Congress had already passed the Clean Air Act (1970), which it supplemented with the Clean Water Act (1972), to authorize urgent measures to address the exterior environment.

Contrast this scenario with the case of indoor air and indoor environments generally. Here, there is not a perceived Shared Common for which the public feels a communal benefit and responsibility. Interior air, whether clean or contaminated, sits within the enclosed space of one’s own home or another discrete building. Typically, a polluter cannot be easily identified and tasked with payment for remediation. If the ambient exterior air is a shared common “owned” by everyone, ownership of indoor air is a far more ambiguous matter.

Consequently, for indoor air, we have had no dramatic moment of recognition such as a river on fire to galvanize public action despite a series of well-publicized serious indoor issues, such as childhood lead poisoning, radon, asbestos, mold, and so on. Furthermore, responsibility for buildings is diffuse, including architects, maintenance personnel, designers, employers, code and building inspectors, occupants, and others. In the absence of a clear-cut villain on whom the burden of correction can be laid, there has been less public support to demand mandates to control indoor pollution.

This is not to say there is neither outrage nor perceived villainy when it comes to indoor air. But in this context, the aggrieved are typically individuals acting for themselves or in small groups rather than an inflamed public acting through the political process. For these reasons, owners of private residences or tenants of buildings typically turn to the courts for redress of specific injury rather than to the legislature for the enactment of broad standards applicable across all occupied spaces. Despite these obstacles, an increasing number of local housing and health code enforcement agencies are adopting local ordinances to improve indoor air quality (Environmental Law Institute 2003), and foundations have increasingly championed “green” communities (Enterprise Foundation 2004).

Indirectly, federal U.S. laws and regulations do address indoor environmental quality. The U.S. EPA administers the Toxic Substances Control Act (1976), the Federal Insecticide, Fungicide, and Rodenticide Act (1996), and the Safe Drinking Water Act (1974), each of which limits the harmful quality of substances brought into the indoor environment, though not indoor air itself. As far back as 1937, the U.S. Housing Act (1937) called for “decent, safe and sanitary housing,” and Congress enacted the Lead-Based Paint Poisoning Prevention Act (1971) and the 1992 Residential Lead Hazard Reduction Act (Title X 1992). The Consumer Product Safety Commission has established regulations prohibiting the sale of lead-based paint and other dangerous consumer products. Most local jurisdictions have health or housing codes that contain prohibitions against broadly defined public health nuisances.

## Successes and Limitations

There are a few examples of successes of government interventions in the indoor environment that have led to dramatic improvements in environmental health, although in each case the improvements have also highlighted the fact that far too many remain at risk and further interventions are needed. Here, we consider the experiences with childhood lead poisoning, radon, and mold.

### Lessons from the lead poisoning prevention experience in the United States

The policy lessons from the lead paint experience provide a framework for addressing other housing-related health hazards. The need for primary prevention (taking action before harm occurs); validated and affordable exposure assessment and hazard control techniques; surveillance; targeted public investment; articulation of a national plan; cost–benefit analyses; enforcement; and policy-relevant scientific research were all at work here.

In one of the clearest examples of the linkage between health and housing, public health officials warned over 100 years ago (Gibson 1904; Turner 1897) against permitting the use of lead paint in housing, a legacy only recently addressed in the United States (Jacobs 1995; Markowitz and Rosner 2002) and in France (Fassin and Naude 2004). Unfortunately, lead paint is still being used in much of the developing world, constituting an emerging housing problem (Alliance to End Childhood Lead Poisoning and Environmental Defense Fund 1994; Clark et al. 2006). Many countries have not identified the prevalence of lead paint hazards in housing (Howson et al. 1996).

Lead poisoning prevention in the United States has emerged as a major public health success story, although childhood lead poisoning still remains a major environmental disease. The number of lead-poisoned children steadily declined in the United States after government action was taken to remove lead from new paint, gasoline, food canning, and most recently from housing, where it presents a hazard. In particular, the removal of lead from gasoline and control of industrial emissions greatly reduced the exposure problem with airborne lead. As a result of these and other measures, the percentage of children 1–6 years of age with blood lead levels ≤ 10 μg/dL declined from 88% in the 1980s to approximately 1.6% (316,000 children) in 1999–2002 (Brody et al. 2005; Meyer et al. 2003). The number of houses with lead paint in the United States has also fallen from 64 million in 1990 to 38 million in 2000 (Jacobs et al. 2002). Although this progress is substantial, it should be tempered by the realization that it took nearly a century to develop the necessary infrastructure to begin to solve the problem, and that far too many children will be poisoned unnecessarily by lead in the coming years unless additional action is taken. Indeed, the United States did not achieve its goal of eliminating childhood blood lead levels > 25 μg/dL by 2000 (Meyer et al. 2003).

How did the progress in preventing lead poisoning happen? What scientific and policy advances made it possible? And what does that experience mean for addressing other housing-related health hazards?

After many years of paralysis, a political consensus emerged in the early 1990s in the United States that the dislocations in the housing market caused by the added expense of making homes lead safe are less important and far less costly than the impact on children’s health and on society. This resulted in the unusual passage of public health and environmental legislation through a housing law (Title X 1992).

Exposure pathway studies revealed that children were most commonly exposed to deteriorated lead-based paint and the contaminated settled house dust and soil it generated (Bornschein et al. 1987; Charney 1983; Lanphear and Roghmann 1997; Lanphear et al. 1998), although the very earliest studies had also suggested the importance of contaminated dust and soil from paint (Gibson 1904). This led to the development of standardized dust-sampling procedures that were significantly correlated with children’s blood lead levels (Lanphear et al.1995). These procedures were subsequently incorporated into health-based exposure standards for paint, dust, and bare soil for housing undergoing remediation, renovation, or repainting (U.S. EPA 2001a).

Previously, the absence of such standards had sometimes resulted in well-intentioned abatement efforts that may have actually increased blood lead levels in some children (Amitai et al. 1987; Aschengrau et al. 1997). The absence of such standard abatement methods contributed to the policy paralysis described earlier. After all, what policymaker would want to implement a procedure, however well intentioned, that exacerbated the problem? These standards also helped to solidify the legal definition of exactly what conditions constituted a lead-based paint hazard in housing (U.S. EPA 2001a) and how they could be controlled (National Center for Healthy Housing 2004).

Congress required that state-of-the-art procedures be published and broadly implemented in subsidized housing and local law (HUD 1995). The creation of standardized procedures helped to create a viable, professional inspectorate and remediation work force, promoted private sector competition on a level playing field, and thus drove down the average cost of lead paint hazard control. These standardized procedures also provided for the prohibition of dangerous forms of lead paint removal, such as use of torches to burn lead paint, abrasive blasting, and power sanding (HUD 1995; Jacobs 1998), although the U.S. EPA has recently proposed to permit such dangerous practices (U.S. EPA 2006).

The data show that the cost of lead hazard control is far less than the monetized benefits. For example, in federally assisted (i.e., subsidized) housing in the United States, the cost of lead hazard control was estimated to be $253 million, but the benefits were estimated to be $1,143 million ($1.1 billion) in the first year of implementing a new lead paint regulation (HUD 1999b). For all high-risk U.S. housing, the incremental cost of eliminating lead-based paint hazards from 2000 to 2010 is $2.3 billion, but the benefits are at least $11.2 billion (Jacobs et al. 2000). In addition, a retrospective study quantified economic benefits from improved worker productivity due to childhood blood lead declines from 1976 to 1999. With discounted lifetime earnings of $723,300 for each 2-year old expressed in year 2000 dollars, the estimated economic benefit for each year’s cohort of 3.8 million 2-year old children ranged from $110 billion to $319 billion (Grosse et al. 2002).

These cost–benefit analyses, together with the development of a viable infrastructure and compelling scientific research, enabled key federal U.S. agencies, including HUD, the CDC, the U.S. EPA, the Department of Justice, and others, to develop a 10-year plan covering 2000–2010 that defined what resources would be needed to eliminate lead paint hazards in houses and to protect children (Jacobs et al. 2000). The plan estimated how many children would be poisoned over the 10-year period if no action was taken and it estimated the costs of not making homes lead safe. From 2000 to 2005, the plan called for a total of $1.15 billion in investment, but only $0.8 billion has actually been appropriated through fiscal year 2005. Unless additional resources are obtained, this deficit is likely to further delay the goal of making housing safe for children by 2010.

### Lessons from the indoor air activities at the U.S. EPA

In 1989 the U.S. EPA established the Federal Interagency Committee on Indoor Air Quality (CIAQ), under Title IV, Section 403(c) of the Superfund Amendments and Reauthorization Act (1986). The purpose of the CIAQ is to coordinate the activities of the federal government on issues relating to indoor air quality. Since the late 1980s, the U.S. EPA has used the statutory authority in Section 103, Subsections (a) and (b) of the Clean Air Act (1970) to establish a nonregulatory program for the indoor environment that analyzes relevant research and communicates indoor air quality risks and steps citizens can take to protect themselves and their families.

The major indoor air quality priorities of the U.S. EPA are the prevention of lung cancer due to radon and the reduction of illness from respiratory and related diseases, such as asthma, in homes and in schools. Here are some highlights of progress to date.

#### Radon

Through 1996, an estimated 880,000 homes had implemented radon-reducing features. These accomplishments led to the prevention of an estimated 285 future premature cancer deaths annually. By the end of 2003, an estimated 1.7 million homes had radon-reducing features (515,000 active mitigations and 1.2 million homes with radon-resistant new construction), preventing 470 future premature cancer deaths annually. In 2004, the number of annual mitigations increased to over 575,000, and the total number of future lives saved increased to 520 annually.

#### Indoor triggers of asthma

Results of the U.S. EPA 2003 National Survey on Environmental Management of Asthma and Children’s Exposure to Environmental Tobacco Smoke (U.S. EPA 2003) indicate that approximately 30% of people with asthma took all the essential actions recommended by the U.S. EPA to reduce exposure to indoor triggers.

#### Schools

U.S. EPA data (U.S. EPA 2005) indicate that 22% of U.S. schools now have an indoor air quality management plan that meets the U.S. EPA standard for effectiveness.

### Implementing a local healthy homes program in Cuyahoga County, Ohio

The high prevalence of poverty, density of children, and substandard housing place children in the Cleveland area at high risk for pediatric diseases related to the home environment. The area has the largest population (1.4 million) and some of the oldest housing stock in Ohio, with 42% of the housing units built before 1950 and nearly half the population classified as very low, low, or moderate income persons (U.S. Census Bureau 2000).

Idiopathic pulmonary hemosiderosis in infants (IPHI) first arose as an issue in Greater Cleveland. A geographic cluster of 10 cases was identified in 1993–1994, and subsequent analysis found an association between water-damaged building materials, mold, and ventilation systems and IPHI (Dearborn et al. 2002). Between 1993 and 2005, 59 infants were diagnosed with IPHI in the Greater Cleveland area, with 16 resultant deaths (Dearborn 2005).

The Pulmonary Hemosiderosis Prevention Program (PHPP) was established by the local public health community in 1996 and supported with funding through public health agencies and local foundations. As a result, 2,874 inspections have been conducted by PHPP over the past decade, and 37% of the housing units were classified as at-risk and requiring some remediation to mitigate water infiltration and environmental cleanup of mold and other potential asthma triggers in the home environment.

Another significant risk factor was a specific forced-air furnace design in which all of the supply air was drawn from the basement space and none from the living space. This resulted in the migration of significant volumes of air from the basement space to occupied areas of the housing unit. Asthma triggers, irritants, pathogens, and other exposure risks that collect in damp basement air were then distributed into breathing air in living spaces.

A study of a Cleveland house containing significant amounts of *Stachybotrys chartarum* on drywall in the basement due to water infiltration and the furnace configuration described above found that air samples collected in the upstairs living areas contained 5-fold increases in *S. chartarum* spores when the furnace and blower were actively circulating in comparison with quiescent conditions (Vesper et al. 2000). Basements often have the highest incidence of water infiltration, water damage, and mold growth of any other room in the housing structure.

A study of high-risk asthmatic pediatric patients who were hospitalized or had recent emergency department or doctor office visits and had water damage/mold within the home was undertaken to study the effects of simultaneously controlling asthma triggers, excess moisture, and lead paint hazard control. The average cost for the interventions (*n* = 104) performed by trained and licensed lead abatement contractors was $5,635, with 56% of the costs being dedicated to mold and moisture control. These interventions provided the opportunity to combine healthy homes interventions and lead hazard control. The mean maximum number of symptom days declined significantly for the intervention group at 10 months (*p* < 0.0001) and 12 months (*p* = 0.053). Emergency department visits and hospitalizations also declined significantly; 11 of 33 subjects in the control group required hospital care, compared with only 1 of 29 cases in the remediation group (*p* = 0.003) (Dearborn 2005).

Briefly, the Cleveland experience shows that local governments can successfully *a*) combine repairs for moisture problems and lead hazard controls with existing housing programs; *b*) integrate funding resources, scientific evaluations, specifications, and remediation; and *c*) achieve programmatic collaboration between local health and housing organizations.

The Cuyahoga County experience shows that local public health programs can respond to poorly understood pediatric diseases related to housing. Although specific legislation is desirable, it is also possible to use existing housing codes and units of local government to implement healthy housing strategies and improve indoor environmental quality.

## Integrating Emerging Science with Policy

All these examples show that government policy development in this field requires a firm scientific foundation. That foundation must include data on specific adverse health effects related to specific housing and building conditions and on interventions that are practical, effective, and do not inadvertently result in further disinvestment in low-income, quality housing, workplaces, schools, and other buildings. In the cases of lead and radon, the scientific research demonstrating significant harm to the public health was particularly compelling and robust.

Despite significant progress, there is still no comprehensive system in the United States that determines what substances and systems should be permitted into buildings and houses during construction, renovation, and maintenance, and whether construction methods effectively prevent naturally occurring toxic substances such as radon from accumulating to dangerous levels. Unfortunately, most efforts to control building-related disease and injury remain largely reactive not preventive. The introduction of materials and construction methods still is done with little regard for adverse health outcomes. Lead paint was introduced into housing with little thought about the potential for lead poisoning. Urea formaldehyde foam insulation, asbestos, and other similar substances are also examples of materials that cost far more to remove or control than they ever saved in reduced construction or maintenance costs or improved durability. Improvements in weatherization and energy conservation initially had similar adverse moisture management consequences due to sealing of building envelopes, with little or no planning included for the interiors to dry out or for proper ventilation (Engvall et al. 2003; Hirsch et al. 2000).

Furthermore, existing research methods remain ill equipped to disentangle specific housing characteristics from a host of confounding variables to discern their effect on disease causation and exacerbation. In many research studies, inadequate housing factors are simply subsumed under “socioeconomic status” and relegated to a covariate at best. We need research that looks at the effect of specific housing and building conditions together with housing and community interventions if we are to move to a more comprehensive, preventive system that anticipates how changes in housing and communities impact physical and mental health. Former U.S. Surgeon General Leroy Burney, best known for his statement regarding the link between cigarette smoking and lung cancer, said that in public health, unlike the law, the suspect is guilty until proven innocent.

A few examples of areas where this type of research is needed are discussed below.

### Asthma and other respiratory conditions, mold, and moisture

In the United States, asthma rates have increased by 73.9% from 1980 to 1996 (Mannino et al. 2002). More evidence is accumulating that factors in the home environment play an important role in sensitizing children to asthma and triggering attacks. Chronic exposure to allergens in the indoor environment from mold, pets, mice and rats, cockroaches, and dust mites is associated with asthma (Breysse et al. 2004). For example, recent exhaustive reviews found there was sufficient evidence to establish a causal link or association between a number of respiratory conditions including asthma (or asthma exacerbation) and the presence of dust mites, cockroaches, fungi and mold, pet dander, tobacco smoke and other substances [National Academy of Sciences (NAS) 2000], and dampness (NAS 2004).

There is also new evidence that housing interventions are indeed effective in reducing the onset and severity of asthma. A large randomized controlled trial in seven U.S. cities enrolled 937 children with atopic asthma. The experimental group received remediation of allergens in the home: provision of mattress and pillow covers; use of a HEPA (high-efficiency particulate air filter) vacuum cleaner; use of a HEPA air purifier if environmental tobacco smoke was present; and pest control if needed. Follow-up showed that the intervention group experienced fewer symptom days for 2 years (*p* < 0.001), and there were significant reductions in dust mite and cockroach allergens, also for 2 years (*p* < 0.001) (Morgan et al. 2004). Another study showed the rate of doctor-diagnosed asthma was 25% in children residing in deteriorated public housing compared with only 8% in other housing (*p* < 0.01). This same trend also was observed in self-reported health status (Howell et al. 2005).

Many other studies have examined the link between allergens in the home and asthma, but the evidence of clinical significance and the cost-effectiveness of home-based interventions needs to be further substantiated (Morgan et al. 2004; NAS 2000).

### Unintentional injuries

Approximately 55% of unintentional deaths among U.S. children 0–19 years of age occur in the home (excluding motor vehicle accidents), and more than 4 million injuries in the home (39%) require a visit to an emergency department, resulting in over 70,000 hospitalizations each year (Nagaraja et al. 2005; Phalen et al. 2005). Falls in the home were the most prevalent mechanism of injury, and falls on stairs were the most common, yet we have relied almost entirely on educational efforts, with little comprehensive assessment of the effect of altering the physical environment. The evidence that does exist shows that reducing the temperature of hot water heaters to prevent scalding, installing window guards in high-rise buildings, installation and maintenance of smoke alarms and carbon monoxide alarms, and installation of cabinet locks and segregation and locking away poisons all have beneficial effects (Breysse et al. 2004).

### Community design, obesity, poverty, and income segregation

The ways in which our communities are geographically arranged also have important health implications that are only now becoming clear. Rates of obesity and diabetes have increased dramatically in the United States (Mokdad et al. 2001). New communities are rarely designed to encourage walking, exercise, and recreation. Suburban developments are more often optimized for automobile transportation, not walking or biking. Unplanned growth of communities (sprawl) is unlikely to be sustainable and has important health consequences, such as obesity and diabetes, that are ripe for further research (Dannenberg et al. 2003; Frumkin et al. 2004).

In a randomized trial known as Moving to Opportunity, families living in high-poverty U.S. public and assisted housing in five cities were given vouchers (subsidies) to move to privately owned housing in low-poverty areas (*n* = 4,248 households). Two control groups included families that continued to live in public or assisted housing in areas with poverty rates exceeding 40%. From 1994 to 2002, the study found significant improvements in adult obesity and mental health (*p* < 0.05) in the group that moved to low-poverty (i.e., mixed-income) neighborhoods. Improvements in asthma were also significant in one of the five cities (HUD 2003). The study showed that adverse health effects are associated with segregating housing by income, and that policies that promote mixed-income communities are likely to have beneficial health outcomes. A review of programs that increase housing choice and promote mobility shows significant public health benefits (Acevedo-Garcia et al. 2004).

### Incremental versus comprehensive policy change

Traditionally, building and housing interventions for health have proceeded from a categorical, substance-specific program design, i.e., lead, radon, mold, and so on. But from a building systems perspective, it is possible to conduct housing interventions that are focused on four major issues and that could be expected to yield multiple health benefits (HUD 1999a). These include moisture, ventilation, settled dust and cleanable surfaces, and education. For example, controlling moisture can be expected to affect mold-induced illnesses, asthma and other respiratory diseases, lead poisoning (because moisture causes paint deterioration), pests, and perhaps others. Improved ventilation can be expected to help control exposures from off-gassing of building materials, combustion products, organic substances, excess moisture, radon, and so on. Settled dust control will have an impact on lead poisoning, pesticide- and asbestos-induced illnesses, and so on.

In short, research and programs should be designed so that the more common incremental, reactive, trial-and-error substance-specific categorical strategies are incorporated into more comprehensive systems that, if improved, will address many individual toxic substances and unhealthy housing factors simultaneously and at lower incremental cost.

The lead poisoning prevention experience is instructive. The U.S. Congress passed the original Lead-Based Paint Poisoning Prevention Act (1971), but because the research, standards, capacity, and other elements described above were not yet adequately developed, it was not until 1992 that the more comprehensive primary prevention approach embodied in Title X was feasible to implement as a national policy. The challenge of developing a comprehensive healthy homes and indoor environment policy remains the key to further progress.

Without an integrated research and policy agenda, the “science of muddling through” (Lindblom 1959) and tackling each issue one-by-one is likely to preclude a comprehensive, preventive, and integrated policy (Quade 1975). Both the comprehensive and incremental approaches are necessary to achieve meaningful policy change, but the comprehensive approach in this field remains largely untapped.

## Conclusion

The key research and policy challenges in this field include the following:

- Improved and lower-cost housing and building hazard measurement techniques.
- Better understanding of the interactions between specific housing and building conditions with physical and mental health outcomes.
- How interactions with confounding variables in the residential setting can be adequately controlled.
- Long-term assessment of the efficacy of integrated housing- and community-based interventions, in addition to assessment of categorical interventions.
- Better understanding of optimum delivery of integrated services and the construction of improved local capacity to provide comprehensive health-related improvements in buildings through improved collaboration between housing and health agencies at all levels of government.
- Making scientific research findings more accessible and more relevant to housing finance, maintenance, and rehabilitation and housing and community development policies.

Relying solely on a substance-by-substance, categorical, trial and error approach (i.e., the incremental approach) is essentially a pessimistic view about the capabilities of citizens, researchers, policymakers, and nations to understand and ultimately make homes, buildings, and communities safe and viable. Moreover, relying solely on the incremental approach creates barriers among the disciplines of public health, housing, environmental science, urban planning, transportation, and others. Instead, we should work to build a multidisciplinary approach to improve the indoor environment.

By producing the science to identify and control hazards, by building the consensus to end the false tradeoff between affordability and health, by accurately describing the costs and hidden benefits, by implementing primary prevention in the indoor environment, and by creating multidisciplinary capacity and know-how at local and national governments, nations can systematically create living environments that will not harm, but actively promote the well-being of their citizens.
